# Biological disturbance of MiR-425 and its application prospects in cardiovascular diseases

**DOI:** 10.3389/fcell.2025.1593241

**Published:** 2025-05-09

**Authors:** Shan Zhou, Bo Han

**Affiliations:** ^1^ Department of Pediatric Cardiology, Shandong Provincial Hospital Affiliated to Shandong first Medical University, Jinan, Shandong, China; ^2^ The Laboratory of Medical Science and Technology Innovation Center (Institute of Biomedical Engineering and Interdisciplinary Studies), Shandong First Medical University, jinan, China

**Keywords:** MiR-425, biological disturbance, cardiovascular diseases, application, gene prediction

## Abstract

MiR-425 is a biological molecule that has potential applications in cardiovascular diseases. It can regulate biological functions by combining with LncRNAs, binding with proteins, and changing the differentiation of immune cells. MiR-425 also has a role as a biomarker of disease. In cardiovascular diseases, it has clinical significance in reducing inflammation and heart repair, inducing angiogenesis, improving the prediction of atherosclerosis, reducing cardiac fibrosis, and regulating atrial natriuretic peptide to affect cardiovascular function. Target gene prediction and KEGG enrichment analysis are also mentioned.

## 1 Introduction

MicroRNAs (miRNAs) are non-coding RNA molecules containing 21 to 23 nucleotides. These molecular entities are ubiquitously distributed across botanical, zoological, and select viral organisms. Such regulatory molecules participate critically in RNA suppression mechanisms and modulate post-transcriptional genetic expression dynamics. These abbreviated genomic modulators orchestrate precise nucleotide complementarity with specific messenger ribonucleic acid transcript fragments. The regulatory mechanisms encompass multiple strategic interventions: fragmenting targeted RNA strands into discrete molecular components, inducing transcript instability through poly (A) tail truncation, or marginally suppressing protein translation processes. Structurally, these genetic regulatory elements demonstrate remarkable similarities to small interfering ribonucleic acid molecules within RNA interference cascades. Notably, distinctive biogenesis pathways differentiate these molecular actors: the compact regulatory sequences emerge from intricate RNA transcript regions capable of forming condensed hairpin configurations, whereas their counterpart interference RNAs originate from extended double-stranded RNA molecular domains (Gan et al., 2022). Genomic computational analyses propose that the *Homo sapiens* genetic landscape potentially harbors approximately 1,900 to 2,300 compact regulatory ribonucleic acid sequences. Nevertheless, rigorous scholarly curation reveals a more conservative estimate, with merely 500 validated genetic elements meeting stringent molecular classification criteria within the authoritative microRNA gene repository (MirGeneDB). MiRNAs are released into body fluids including blood (Fan et al., 2025), urine (Wang et al., 2025), sweat (Karvinen et al., 2020), Human Breast Milk (Yi and Kim, 2021), Saliva (Urbizu et al., 2023), Tear (Altman et al., 2023) and cerebrospinal fluid (Noh et al., 2025) and have the potential to be available as biomarkers in a number of diseases.

Computational genomic investigations suggest that these compact regulatory ribonucleic acid molecules potentially modulate approximately 60% of genetic elements within human and mammalian systems (Hua et al., 2023). Phylogenetic analyses reveal substantial evolutionary conservation, indicating profound biological significance. Illustrative evidence emerges from comparative molecular studies, where identical genetic regulatory sequences have been systematically identified across divergent species, such as *H. sapiens* and murine cleft lip and palate developmental models, underscoring their critical functional roles (Yoshioka et al., 2021). The molecular landscape of these genetic modulators traces its initial scientific discovery to the nascent stages of the 1990s, though comprehensive recognition of their distinct regulatory mechanisms emerged during the early millennium. Subsequent comprehensive investigations unveiled intricate expression patterns across diverse cellular environments and tissue-specific contexts, demonstrating multifaceted contributions to organismal development and complex biological processes. Emerging translational research increasingly implicates aberrant expression patterns in pathological states, with innovative therapeutic strategies currently under rigorous investigational protocols (Diener et al., 2022).

The biogenesis of microRNAs occurs via two principal routes: the canonical and non-canonical pathways (Ergin and Çetinkaya, 2022). Canonical Pathway: Genetic regulatory sequences undergo initial transcriptional processes mediated by RNA polymerase II, generating primary molecular precursors. These nascent transcriptional units subsequently experience nuclear processing via a sophisticated molecular machinery, encompassing the RNA-binding protein DiGeorge Syndrome Critical Region 8 (DGCR8) and the specialized ribonuclease III enzymatic component Drosha. Subsequent cytoplasmic translocation facilitates further molecular refinement through RNase III endonuclease intervention, culminating in mature duplex formation. Ultimately, selective strand incorporation into Argonaute (AGO) protein complexes establishes a regulatory silencing mechanism targeting specific messenger RNA transcripts. Alternative Pathways: Divergent biogenesis strategies predominantly include Drosha/DGCR8-independent and Dicer-independent molecular mechanisms. The intronic processing pathway enables direct precursor generation from messenger RNA interstices, circumventing traditional Drosha-mediated cleavage. Conversely, the Dicer-independent route involves specialized AGO2-mediated maturation of abbreviated endogenous short hairpin RNA transcripts, which are structurally incompatible with conventional Dicer processing protocols (Miao et al., 2024).

These two distinct isoforms emerge from opposite arms of the same pre-miRNA hairpin during the biogenesis process. The -5p variant originates from the 5′arm, while the -3p variant derives from the 3′arm. Despite sharing a common precursor, these isoforms possess different seed sequences that target distinct mRNA populations, thereby regulating separate gene networks and potentially exerting divergent or even opposing biological effects. In pathological contexts, miR-425-5p generally demonstrates oncogenic properties across multiple cancer types, promoting proliferation, invasion, and chemoresistance by targeting tumor suppressors like PTEN (Xiao et al., 2019) and CYLD (Yan et al., 2017). Contrastingly, miR-425-3p exhibits a more complex profile, functioning as a tumor suppressor in certain cancers (Zhang et al., 2022). In inflammatory and metabolic disorders, these isoforms can regulate different aspects of disease progression, with -5p often involved in cell survival pathways while -3p modulates inflammatory responses (Gao et al., 2024; Przybyciński et al., 2025).

The tissue-specific expression patterns and context-dependent functions of these isoforms highlight the complexity of miRNA-mediated regulation. Their differential expression ratios may serve as valuable diagnostic and prognostic biomarkers, while their distinct regulatory networks offer potential therapeutic targets. Understanding the isoform-specific behaviors provides critical insights into disease mechanisms and may inform more precise therapeutic strategies for various pathological conditions.

Contemporary molecular investigations have unveiled an expansive repertoire of regulatory mechanisms beyond traditional gene expression suppression. Emerging research demonstrates that microRNAs exhibit sophisticated multifunctional capabilities. Preliminary transcriptional units potentially undergo translational processes, generating bioactive peptide configurations capable of executing diverse physiological roles through cytoplasmic interactions and ribosomal recognition (Goto and Suga, 2021). Furthermore, these molecular regulators establish intricate protein complexes with Argonaute-associated molecular machinery, transcending conventional degradative pathways. Their functional plasticity enables simultaneous engagement with alternative protein networks, facilitating nuanced regulatory cascades that extend beyond classical genetic modulation. Such sophisticated molecular strategies introduce unprecedented complexity and functional diversity to the regulatory landscape, challenging conventional understanding of genetic information processing. These sophisticated molecular mechanisms significantly expand our comprehension of genetic regulatory networks, revealing a multidimensional framework of post-transcriptional control that surpasses traditional conceptual boundaries. The emergent molecular paradigm underscores the remarkable adaptability and functional versatility of these compact genetic modulators.

In the development, progression, and regression of cardiovascular system miRNAs play a critical role (Zhou et al., 2018), such as myocardial infarction, hypertension, and atherosclerosis. The study of microRNAs in cardiovascular diseases has opened up new ways to develop diagnostic and treatment strategies (Michell and Vickers, 2016; Kontidou et al., 2023). In cardiovascular diseases, microRNAs have been discovered to play a role in controlling many different biological activities, such as maintaining mitochondrial function (Roiz-Valle et al., 2023), inflammation (Emmi et al., 2022), oxidative stress (Ibáñez-Cabellos et al., 2023), apoptosis (Diakos et al., 2010), angiogenesis (Pencheva et al., 2012), and lipid metabolism (Elsakka et al., 2023). By regulating these processes, microRNAs can lead to the development or worsening of cardiovascular diseases. MicroRNAs also have potential to be used as diagnostic (Wang and Zhang, 2020) and therapeutic tools (Poller et al., 2018). Researchers have identified specific microRNAs that are dysregulated in cardiovascular diseases, and these microRNAs could be used as biomarkers for early diagnosis or disease monitoring. Additionally, by using drugs or gene therapies that target microRNAs that are not functioning properly (Kara et al., 2022), we may be able to treat heart and blood vessel problems more effectively in the future (Li and Rana, 2014).

In this review, we focus on microRNA-425 (miR-425), examining its biological dysregulation and therapeutic potential in cardiovascular diseases. By synthesizing current evidence, we highlight its role in disease pathogenesis, including aberrant signaling pathways and cellular dysfunction, and explore its emerging applications as a diagnostic biomarker and therapeutic target in cardiovascular medicine.

## 2 The biological perturbation of MiR-425

### 2.1 Combined with LncRNAs to regulate biological function

Recent studies have found that miR-425 can work together with long non-coding RNAs to have additional control over various biological processes. This interaction between miR-425 and lncRNAs plays a role in regulating these functions within cells or organisms.

LncRNAs are long non-coding RNAs that have various functions in gene regulation, chromatin remodeling, and cellular differentiation. They are also involved in some diseases such as cancer, neurodegeneration, and cardiovascular disorders. One study found that a lncRNA called HHIP-AS1 promotes tumorigenicity by stabilizing a protein complex called dynein complex 1 in cancer prompt by SHH signaling pathway (Bartl et al., 2022). HHIP-AS1 binds directly to the mRNA of DYNC1I2, a component of dynein complex 1, and protects it from reduction by miR-425-5p. MSC-AS1 is a lncRNA that suppresses ovarian cancer by binding to miR-425-5p (Zhao et al., 2021). It negatively regulates miR-425-5p expression and affects its downstream targets such as CCND2 and BCL2. A third study found that miR-425-5p reduce two lncRNAs, MALAT1 and TUG1, and suppresses tumorigenesis (Yang et al., 2019). The latest study demonstrated that miR-425-5p modulates viral myocarditis through its interaction with lncRNA NEAT1 (17). The long non-coding RNA HCG22 emerged as a critical oncogenic modulator in papillary thyroid carcinoma (PTC), exerting profound influences on cellular dynamics through suppressive interactions with miR-425-5p. Mechanistic investigations revealed that HCG22 substantially enhanced neoplastic progression by modulating proliferative capacities and facilitating metastatic potential through targeted miRNA downregulation (Cao et al., 2024). These lncRNAs are one type of competing endogenous (ceRNAs) that can regulate miR-425-5p by this mechanism. Thus, miR-425 and lncRNAs form a complex interaction network that modulates cell proliferation in various cancers.

In addition to cell proliferation and migration, miR-425 has also been shown to interact with lncRNAs to regulate apoptosis, a process of programmed cell death. For example, miR-425-5p and the lncRNA MSC-AS1 have been found to interact in SKOV3 and A2780 cancer cells. When miR-425-5p is upregulated, it counteracts the overexpression of MSC-AS1. High levels of MSC-AS1 inhibit cell proliferation and promote cell apoptosis, highlighting the importance of their regulation in these cancer cell lines (Zhao et al., 2021). Another study found that lncRNA SNHG7 regulated the expression of TRAF5 by sponging miR-425-5p. TRAF5 is a key mediator of the NF-κB pathway, which plays a critical role in inflammation and apoptosis (Zhang et al., 2021). Lastly, during ischemic stroke, the long non-coding RNA Snhg8 plays a crucial role in reducing microglial inflammation. Snhg8 acts as a competitive endogenous RNA by binding to and sequestering miR-425-5p, the function is protective (Tian et al., 2021). Overall, these studies suggest that miR-425 and lncRNAs can work together to regulate biological functions, for example cell expansion, migration, and apoptosis. Further research into these interactions can promote the advancement of new therapies for disease.

### 2.2 Binding with protein to regulate biological function

Another way in which miR-425 regulates biological function is by binding to specific proteins and modulating their expression or activity. MiR-425-5p has been found to improve liver damage. It Binding RIP1 mRNA to subdued the expression. Liver damage, RIP1-mediated necroptosis, IL-1β, and TNF-α were suppressed by miR-425-5p ago-miR but further aggravated by miR-425-5p antagomiR (Gu et al., 2020)). MiR-425-5p can binding TNF, ANXA2 in MSC cell. In addition, miR-425-5p enhanced osteoporosis in mice. MiR-425-5p might serve as a potential therapeutic target for the treatment of osteoporosis (Chen et al., 2021). According to this essay (Qi et al., 2019) MiR-425 is a novel strong regulator of adipogenesis and adipolysis in adipocytes. The literature (Tokarski et al., 2022) suggests that miR-425 directly regulate the expression of Hepatocyte growth factor (HGF) under hypoxia. HGFA is stimulate the proliferation and migration of endothelial cells. Come to conclusion that miR-425 is a crucial regulator in HGFA-HGF-c-Met signaling pathway. MiR-425-5p plays a pivotal role in modulating gemcitabine resistance through its intricate interactions within the MEF2A/SNHG16/NOTCH2 signaling axis, potentially offering novel insights into therapeutic strategies for chemoresistant malignancies (Zhou X. et al., 2024). MiR-425-5p emerges as a critical molecular regulator of bovine mammary epithelial cell (BMEC) proliferation, exerting its functional influence through precise modulation of the TOB2 signaling pathway. By targeting this specific molecular mechanism, the microRNA significantly contributes to cellular expansion and potentially provides insights into mammary gland development and function (Li et al., 2024).

These studies indicate miR-425 regulates cellular biological functions by binding with many target proteins. These proteins exhibit biological functions involved in apoptosis, angiogenesis, and metastasis regulation. The binding of miR-425 can directly inhibit the translation or degradation of these proteins, affecting cellular biological functions. These findings further demonstrate the important role of miR-425 in cellular biological processes and provide a new therapeutic approach to modulate these processes by regulating the of miR-425.

### 2.3 Change the differentiation of immune cells to regulate biological functions

In osteoarthritis patients, miR-425 is involved in leukocyte migration, regulation of mitogen-activated protein (MAP) kinase tyrosine/serine/threonine phosphatase activity, interleukin-17 signaling pathway, and osteoclast differentiation, and can exert a strong influence on immune cell infiltration (Hua et al., 2022).

In Inflammatory bowel disease (IBD) (Yang et al., 2018), miR-425 play a role in the development of Th17 cells by targeting Foxo1. It means that increasing the levels of miR-425 (a type of small RNA molecule) in inflammatory bowel disease (IBD) can lead to the production of harmful Th17 cells by reducing the activity of a protein called Foxo1. The research has demonstrated that miR-425 hinders the growth of chronic lymphocytic leukemia (CLL) cells by controlling the activity of the Bruton’s tyrosine kinase/phospholipase Cγ2 signaling pathway (Chen et al., 2020). Another literature states that (Wu et al., 2021) when miR-425 is high-expressed in diffuse large B cell lymphoma (DLBC) cells, it can lead to cells grow and divide. This can also increase their ability to form colonies. The differential association between the miR-425 and macrophage might be crucial for Colorectal cancer (CRC) (Ng et al., 2022). The article display that there was positive correlation between miR-425 and macrophages in CRC.

## 3 The role of biological disease markers

In neurological disorders (Manna et al., 2021) and diabetic retinopathy (Liu X. et al., 2022), miR-425 can also serve as a disease marker. Notably, miRNAs can be found in different bodily fluids like blood, saliva, and urine. This characteristic makes them valuable as biomarkers for the detection, prognosis, and treatment of various diseases.

One specific example of miR-425 in cancer is in metastatic prostate cancer (Rode et al., 2021; Rana et al., 2022). A study found that miR-425-5p as an exosomal biomarker. Research has indicated that miR-425 shows varying levels of expression in extracellular vesicles derived from both normal cells and prostate cancer cells. A study suggests that miR-425-5p can discriminated Progressive Supranuclear Paralysis from Parkinson’s disease (Manna et al., 2021). A recent study found that serum miR-425-5p levels showed a gradual increasing trend in the healthy control group, the diabetic mellitus patients without diabetic retinopathy, and diabetic retinopathy patients. Moreover, the levels of miR-425-5p in proliferative DR (PDR) patients were elevated than that of non-PDR (NPDR) patients (Liu X. et al., 2022). Emerging evidence suggests that miR-425-5p, a pivotal microRNA implicated in programmed cell death mechanisms, demonstrates notable predictive potential in stratifying patients with esophageal squamous cell carcinoma (ESCC). The molecular signature of this specific miRNA offers promising insights into disease progression and potential diagnostic strategies (Zhidan et al., 2024).

## 4 The clinical significance of MiR-425 in cardiovascular diseases

### 4.1 Reduces inflammation and heart repair

It has been reported that miR-196a-5p and miR-425-5p, which are found in exosomes derived from adipose-derived stem cells (ASC), can influence biological processes after a myocardial infarction. Both miRNAs were shown to prevent mitochondrial dysfunction and increase angiogenesis (Paiva and Agbulut, 2017), and polarize macrophages toward M2 (de Almeida Oliveira et al., 2022). A study found that miR-425 directly target receptor-interacting protein kinase 1 (RIPK1), and overexpression of miR-425 or knockdown of RIPK1. So miR-425 can become a promising therapeutic agent for treating heart injury by being activated by IR (Guo et al., 2020). Another study suggests that miR-425-3p is lowly expressed in viral myocarditis. Overexpression of miR-425-3p was found to improve cardiac function, alleviate pathological conditions. Additionally, miR-425-3p was found to bind to TGF-β1 and suppress its expression. These results suggest that overexpression of miR-425-3p can inhibit myocardial inflammation in mice with viral myocarditis (Li et al., 2021). In a research study (Chaudhari et al., 2023), the global expression patterns of miRNAs were analyzed in mouse ventricular tissue samples collected at different stages of postnatal development: postnatal day 1 (P01), P04, P09, and P23. The study discovered that miRNAs have a significant role in the development of the heart after birth and in the process of cardiac regeneration.

In summary, the findings from these studies indicate that miR-425 could be a promising therapeutic option for various treatments of heart disease by reducing inflammation (Zapata-Martínez et al., 2023), cardiac regeneration and promoting heart repair. However, additional research is necessary to gain a comprehensive understanding of how miR-425 functions and to investigate its potential clinical uses.

### 4.2 Induce angiogenesis and improve the prediction of atherosclerosis

Understanding the intricate mechanisms underlying angiogenesis is essential for developing novel therapeutic strategies to target angiogenic disorders. Recently, miRNAs have been widely acknowledged for their involvement in a wide range of biological processes, including angiogenesis (Kir et al., 2018). Among these miRNAs, miRNA-425-5p has gained attention for its involvement in the complex orchestration of angiogenesis. Emerging evidence suggests that miRNA-425-5p acts as a crucial regulator of angiogenesis by modulating the activity of specific target genes in different stages of angiogenic process. Such as Spred1, SEPT7, and PDGFRA genes (Ferguson et al., 2018; Liu et al., 2021; McGeary et al., 2019). In a study, sodium arsenite (NaAsO2) was found to impede the formation of new blood vessels (angiogenesis). However, when miR-425-5p was over-expressed, the anti-angiogenic effects caused by NaAsO2 were reversed (Gao et al., 2016). Certain microRNAs, including miR-425-5p, were found to be abundant in exosomes derived from epidermal stem cells (EPSC-Exos). These microRNAs played a crucial role in suppressing the transformation of fibroblasts into myofibroblasts by reducing the expression of TGF-β1 in dermal fibroblasts. In summary, this study identified a new function of EPSC-Exos-specific microRNAs, indicating that miR-425-5p could be utilized as a potential strategy to prevent scar formation and angiogenesis during wound healing in clinical applications (Duan et al., 2020). Research (Kim et al., 2022) findings indicate that miR-425-5p, which is classified as an onco-miRNA, was detected within small extracellular vesicles (sEVs) that rely on syntenin-1 for their formation. These sEVs were observed to have an impact on cancer cell migration and angiogenesis.

Abnormal expression of miRNAs has been linked to the development and advancement of atherosclerosis (Sum and Brewer, 2023). A recent study (Ormseth et al., 2021) investigated the potential of miRNA-425 as a predictive biomarker for coronary atherosclerosis. The study included a group of rheumatoid arthritis (RA) patients with and without coronary atherosclerosis, as confirmed by angiography. Plasma samples were collected from the participants. The study findings demonstrated a significant increase in plasma levels of miRNA-425 among rheumatoid arthritis (RA) patients with coronary atherosclerosis, in comparison to those without the condition. Additionally, there was a positive correlation observed between the activity of miRNA-425 and the severity of coronary atherosclerosis, as assessed by angiographic findings. This suggests that miRNA-425 may contribute to the development of coronary atherosclerosis in the context of rheumatoid arthritis. Furthermore, the study assessed the diagnostic performance of plasma miRNA-425 for predicting coronary atherosclerosis in RA. ROC curve analysis show that miRNA-425 had good discriminatory power. These findings suggest that measuring plasma levels of miRNA-425 could enhance the accuracy of predicting coronary atherosclerosis in individuals with rheumatoid arthritis.

### 4.3 Reduces cardiac fibrosis

In a specific study (Wang et al., 2023), a comprehensive analysis identified a total of 18 miRNAs targeted in human atrial appendage tissues. In another study (Wei et al., 2022), the researchers examined the involvement of miR-425-5p in atrial fibrillation (AF) and investigated its effects on atrial fibrosis and remodeling. The study revealed that miR-425-5p level was significantly downregulated in AF patients compared to those without AF. Further mechanistic investigations demonstrated that miR-425-5p inhibits the expression of CREB1 in atrial cells. CREB1 is a transcription factor involved in various cellular processes, including fibrosis and remodeling. The decreased expression of miR-425-5p in atrial fibrillation (AF) results in elevated levels of CREB1, promoting atrial fibrosis and remodeling. Functional experiments utilizing animal models of AF confirmed the regulatory role in atrial fibrosis and remodeling. Overexpression resulted in decreased fibrosis and improved atrial electrical conduction properties, while downregulation had the opposite effect. Complementary investigative evidence demonstrates that fibroblast-originated compact regulatory ribonucleic acid sequences (miR-425-5p) attenuates transverse aortic constriction-mediated cardiac dysfunction, positioning this molecular marker as a promising diagnostic and interventional strategy for managing advanced cardiac insufficiency pathologies (Zhou H. et al., 2024).

In summary, above results establishes miR-425-5p as a negative regulator of atrial fibrosis and a promoter of atrial remodeling. It suggests that the decreased level of miR-425-5p in AF contributes to the progression of atrial fibrosis and highlights the importance of targeting miR-425-5p and its downstream target CREB1 as potential therapeutic strategies for AF. By modulating miR-425-5p levels, it may be possible to mitigate atrial fibrosis and prevent the adverse remodeling processes associated with AF, ultimately leading to improved management and treatment of this prevalent cardiac arrhythmia. Further research is warranted to explore the translational potential of miR-425-5p as a therapeutic target in AF.

### 4.4 Regulating atrial natriuretic peptide affects cardiovascular function

Atrial natriuretic peptide (ANP) is essential for maintaining cardiovascular equilibrium by controlling blood pressure, fluid balance, and electrolyte levels. Recent studies have identified miR-425 as a negative regulator of ANP (Arora et al., 2013). ANP is synthesized and released in response to increased cardiac wall tension, which occurs during conditions such as hypertension, heart failure, or volume overload. Once released, ANP acts on target tissues, primarily the kidneys, to induce natriuresis (sodium excretion) and diuresis (increased urine production), thereby reducing blood volume and blood pressure. ANP also inhibits the secretion of renin and aldosterone, further contributing to blood pressure regulation. In a study (Vandenwijngaert et al., 2018) conducted on rat models of heart failure, an elevated expression of miR-425 was observed in hearts experiencing heart failure. Overexpression of miR-425 resulted in decreased ANP expression and secretion. Similar findings were observed in human cardiac tissues, suggesting the conservation of miR-425-mediated regulation across species. The dysregulation of ANP expression is associated with various cardiovascular diseases. The identification of miR-425 suggests a potential therapeutic target for these conditions. Modulating miR-425 levels or targeting its downstream effectors may offer a novel approach to enhance ANP production and its beneficial effects on cardiovascular function. MiRNA-425 has emerged as a key regulator of ANP expression and secretion. Its upregulation in heart failure and its ability to decrease ANP levels indicate its involvement in cardiovascular pathology. Understanding the mechanisms underlying miR-425-mediated regulation of ANP may pave the way for the development of novel therapeutic strategies targeting the ANP pathway. Additional research is required to fully investigate the extent of miR-425 modulation in cardiovascular physiology and its clinical implications in the management of cardiovascular diseases.

## 5 Target gene prediction and KEGG enrichment analysis

**FIGURE 1:**
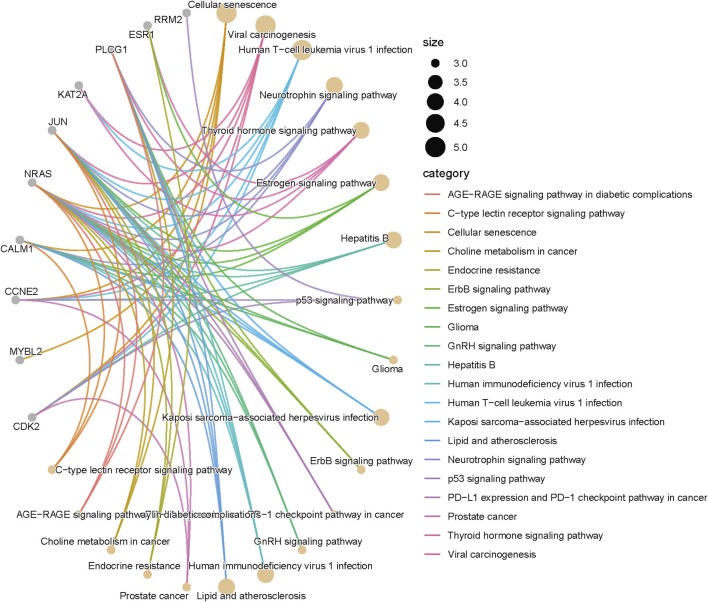
Target Gene prediction and KEGG enrichment analysis.

Target gene prediction and KEGG enrichment analysis can be conducted to gain insights into the function and pathways influenced by miRNAs. Here, we will outline the process of target gene prediction and the subsequent KEGG enrichment analysis for miR-425. Several computational tools and databases are available for predicting miRNA target genes. Some commonly used tools include TargetScan, miRanda, and miRDB. These tools utilize algorithms that consider sequence complementarity between the miRNA and the mRNA, as well as other features such as conservation across species and the presence of 3′UTR. To predict target genes for miR-425, we input miR-425 into TargetScan and miRDB, take the intersection, and predict a total of 247 target genes. We then used the R package “clusterProfiler, enrichplot, ggplot2, pathview, ggnewscale, DOSE” for KEGG enrichment analysis and obtained Figure 1.

## 6 Conclusion and perspectives

As illustrated in Figure 1 and Table 1, miR-425 may exert its biological functions by interfering with the expression of key genes in several common pathways. Notably, the relatively large circle in the Cellular senescence pathway indicates that miR-425 likely plays a significant role in this pathway among those depicted. Through literature review, we found confirmatory evidence of this relationship in a study demonstrating that miR-425-5p inhibits Crebzf to regulate oocyte senescence via chromatin modification (Jueraitetibaike et al., 2024). Although Crebzf was not identified among the key genes shown on the left side of Figure 1, the gene network presented provides a broad spectrum of potential research targets. For instance, Figure 1 depicts a connection between NRAS and Cellular senescence. Our literature analysis revealed that titration of RAS alters senescence states and influences tumor initiation, with NRAS being one of the most common members of the RAS family (Chan et al., 2024). Integrating the findings from Figure 1 with these two studies suggests a hypothesis that miR-425 may influence disease progression through NRAS-mediated cellular senescence. In this manner, Figure 1 serves as a valuable source of research inspiration.

**TABLE 1:** MiR-425 in cardiovascular diseases.

Roles	Cardiovascular diseases	References
Reduces inflammation	After myocardial infarction, polarize macrophages toward the anti-inflammatory M2 immunophenotype directly target receptor-interacting protein kinase 1 (RIPK1) attenuate inflammation	de Almeida Oliveira et al. (2022) (Guo et al., 2020)
	Interaction with TGF-β1 reduce viral myocarditis decrease Interaction with lncRNA NEAT1 reduce viral myocarditis decrease	Li et al. (2021) (Gao et al., 2024)
Induction of angiogenesis	Target cerebral cavernous malformation 3 (CCM3)	Gao et al. (2016)
	Prevent scar formation and angiogenesis	Duan et al. (2020)
	As onco-miRNA promote cancer cell migration and angiogenesis	Kim et al. (2022)
Prediction of diseases	Predictive biomarker for coronary atherosclerosis in patients with rheumatoid arthritis markers for diagnosing heart failure	Ormseth et al. (2021) (Zheng et al., 2020)
Reduces cardiac fibrosis	Inhibits the expression of CREB1 in atrial cells promoting atrial fibrosis and remodeling Alleviates cardiac remodelling in heart failure	Wei et al. (2022) (Zhou et al., 2024b)
Regulating ANP	Negative regulator of ANP	Arora et al. (2013), Vandenwijngaert et al. (2018)

Through the above sections, miR-425 has been identified as a significant contributor to the development and progression of cardiovascular diseases, including heart failure, hypertension, atherosclerosis, and cardiac remodeling. Its dysregulation contributes to the progression of these diseases through its effects on various molecular pathways involved in cardiac function, vascular remodeling, and endothelial dysfunction.

The dysregulation of miR-425 expression in cardiovascular diseases opens up new avenues for its potential use as a diagnostic biomarker. Circulating levels of miR-425 in plasma or serum have shown correlations with disease severity, treatment response, and clinical outcomes. Utilizing miR-425 as a non-invasive biomarker could enhance early detection, risk stratification, and monitoring of cardiovascular diseases, ultimately leading to improved patient management and outcomes. Furthermore, targeting miR-425 holds promise for developing novel therapeutic strategies. Inhibition of miR-425 using antimiR oligonucleotides or viral vectors expressing competing endogenous RNAs (ceRNAs) has shown promising results in preclinical studies. By suppressing the activity of miR-425, it is possible to improve cardiac function, attenuate cardiac remodeling, and ameliorate hypertension. However, it is crucial to further optimize the delivery methods, ensure target specificity, and evaluate the long-term safety and efficacy of miR-425-based therapies before translation into clinical applications. The role and prospects of miR-425 in cardiovascular disease highlight the growing importance of understanding the intricate regulatory mechanisms involved in cardiac pathophysiology. Further research is needed to elucidate the precise molecular targets and signaling pathways influenced by miR-425, as well as to investigate potential synergistic effects with existing therapeutic approaches.

In brief, miR-425 represents a promising area of study for researchers and clinicians in the field of cardiovascular medicine. Continued investigations into its role, diagnostic potential, and therapeutic applications will deepen our understanding of cardiovascular diseases (Mota et al., 2023) and potentially lead to the development of innovative approaches for disease management and treatment.
